# Attomolar Detection of Botulinum Toxin Type A in Complex Biological Matrices

**DOI:** 10.1371/journal.pone.0002041

**Published:** 2008-04-30

**Authors:** Karine Bagramyan, Jason R. Barash, Stephen S. Arnon, Markus Kalkum

**Affiliations:** 1 Immunology Division, Beckman Research Institute of the City of Hope, Duarte, California, United States of America; 2 Infant Botulism Treatment and Prevention Program, California Department of Public Health, Richmond, California, United States of America; University of California Merced, United States of America

## Abstract

**Background:**

A highly sensitive, rapid and cost efficient method that can detect active botulinum neurotoxin (BoNT) in complex biological samples such as foods or serum is desired in order to 1) counter the potential bioterrorist threat 2) enhance food safety 3) enable future pharmacokinetic studies in medical applications that utilize BoNTs.

**Methodology/Principal Findings:**

Here we describe a botulinum neurotoxin serotype A assay with a large immuno-sorbent surface area (BoNT/A ALISSA) that captures a low number of toxin molecules and measures their intrinsic metalloprotease activity with a fluorogenic substrate. In direct comparison with the “gold standard” mouse bioassay, the ALISSA is four to five orders of magnitudes more sensitive and considerably faster. Our method reaches attomolar sensitivities in serum, milk, carrot juice, and in the diluent fluid used in the mouse assay. ALISSA has high specificity for the targeted type A toxin when tested against alternative proteases including other BoNT serotypes and trypsin, and it detects the holotoxin as well as the multi-protein complex form of BoNT/A. The assay was optimized for temperature, substrate concentration, size and volume proportions of the immuno-sorbent matrix, enrichment and reaction times. Finally, a kinetic model is presented that is consistent with the observed improvement in sensitivity.

**Conclusions/Significance:**

The sensitivity, specificity, speed and simplicity of the BoNT ALISSA should make this method attractive for diagnostic, biodefense and pharmacological applications.

## Introduction

Botulinum neurotoxins (BoNT)s are the most poisonous substances known [1], [2]. They cause the human illnesses of infant [3]–[5], wound [6], foodborne [7] and iatrogenic botulism [8]–[11], but are also used to treat a variety of medical conditions [12], [13]. The potential abuse of BoNT in bioweapons is feared [1], [14]. The enormous potency of the toxin is reflected by its estimated human lethal *i.v.* dose of only 1-2 ng/kg body weight [1], [2]. Hence, the detection of low, but nonetheless dangerous amounts of BoNT in complex clinical specimens or foods represents an extreme analytical challenge.

As produced by *Clostridium* bacteria, BoNT is present within ∼300, 500 or 900-kDa protein complexes together with non-toxic components, known as neurotoxin associated proteins (NAPs) [15]–[20]. Seven structurally distinct serotypes of BoNT (A to G) have been discovered. The neurotoxin itself is a 150-kDa zinc-binding metalloprotease (holotoxin) that is endogenously cleaved into a 100-kDa heavy and a 50-kDa light chain that are connected by a reducible disulphide bond [21] and by a belt-like extension of the heavy chain that loops around the light chain [22]. The catalytic site is located on the light chain [23]. Reduction of the chain-bridging disulphide bond allows for chain separation and exposure of the catalytic site, which enhances the toxin's activity [22], [24], [25]. The phenomenal potency of BoNT results from its ability to enzymatically cleave one or more of the three SNARE proteins that are involved in fusing acetylcholine-containing synaptic vesicles with the terminal motor neuron membrane and trigger muscle contraction [21], [26]–[28].

The definitive diagnosis of botulism requires the detection of BoNTs in a clinical specimen. The most commonly used method to accomplish this task is the live-mouse bioassay [29]–[31] that can detect as little as 10 pg of BoNT holotoxin [32], and, because of its sensitivity, simplicity and robustness, it is considered the “gold-standard”. Other, generally faster, methods for detecting BoNT include various enzyme-linked immunosorbent assays (ELISAs) [30], a cantilever-based micromechanosensor [33], protein-based fluorescence resonance energy transfer (FRET) sensors [34], enzyme-amplified protein micro-arrays [35], mass spectrometric assays [36]–[39], immuno-PCR detection [40], and recently, a real-time PCR-based assay that utilizes reporter DNA-filled liposomes that bind to immobilized BoNT/A via gangliosides [41], [42]. **Table 1** lists detection limits and the types of samples for which BoNT assays were demonstrated. Most assays, except for a PCR-based one, are unable to detect less than 1 pg/mL BoNT in a complex sample, such as a body fluid.

**Table 1:** Performance of existing botulinum toxin assays.

Test method	Demonstrated for Sample Type	Detection Limit (fg/mL)	Assay Time
Cantilever-based micromechanosensor [33]	Sample buffer [33] ^a^	4×10^8^	15 min.
Mass spectrometry [36]–[39]	milk, serum, stool extract^b^	320,000	<4 hrs
ELISA [30]	liquid and solid foods, serum^c^	60,000	6–8 hrs
ELISA-HRP [43]	therapeutic preparations^d^	9,000	4–6 hrs
Mouse assay [29]	foods, serum, stool^e^	∼6,000	typically 48 hrs
Enzyme-amplified protein microarray immunoassay [35]	blood, plasma^f^	1,400	<10 min.
Immuno-PCR [40]	carbonate buffer^g^	50	4–6 hrs
Liposome PCR assay [41], [42]	deionized water^h^	0.02	6 hrs
ALISSA (this study)	serum, milk, carrot juice, gelatin phosphate diluent^h^	0.5	2–3 hrs

Reported for: ^a^BoNT type B light chain; ^b^BoNT complex types A-G; ^c^BoNT complex types A, B, E, and F; ^d^BoNT purified from culture supernatant of *C. botulinum* type A (A7272); ^e^Spores and toxin of *C. botulinum* type A, E, and spores of *Clostridium sporogenes*; ^f^fragment of the heavy chain of BoNT type A; ^g^BoNT types A, B, and E noted as “purified to apparent homogenous from different strains of *C. botulinum*”; ^h^BoNT/A.

Here we report a simple method, termed the BoNT Assay with a Large Immuno-sorbent Surface Area (ALISSA), that uses common laboratory equipment and commercially available reagents. The BoNT ALISSA can detect less than 0.5 fg of the toxin (500-kDa BoNT/A complex) in 1 mL of serum, milk, carrot juice or gelatin phosphate (GP)-diluent within 2.5 hours. We present a detailed protocol and explain how we optimized the ALISSA and defined its kinetic parameters. Our assay has the potential to significantly improve the diagnosis of botulism and could serve to protect humans in biomedical and bio-defense scenarios.

## Results

### Assay Design

**Figure 1:**
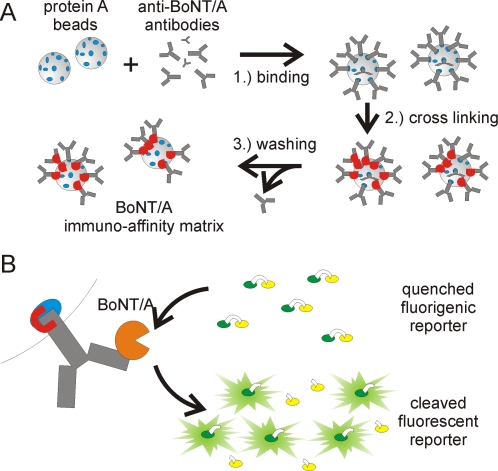
The ALISSA principle. (A) Synthesis scheme for the immuno-affinity matrix for BoNT-enrichment. Protein A-agarose beads are coupled to affinity-purified anti-BoNT antibodies. Disuccinimidyl suberate (DSS) is used to crosslink the FC domain of the antibodies to protein A. Non-crosslinked antibodies are removed by stringent washing. (B) Immobilized BoNT/A cleaves a quenched FRET pair, provided in form of a fluorogenic peptide substrate, thereby releasing the fluorophore from the quencher, which restores fluorescence.

BoNT/A ALISSA consists of two main steps. First, BoNT/A is captured and enriched on a bead-based immuno-affinity matrix and molecules that bind the matrix non-specifically are washed away. Second, the enzymatic activity of the immobilized BoNT/A is determined by cleavage of a specific fluorogenic substrate. Our enrichment matrix consists of protein A-conjugated sepharose beads to which we coupled and crosslinked polyclonal anti-BoNT/A antibodies (Figure 1A). These immobilized antibodies capture BoNT/A molecules with high affinity and they do not inhibit BoNT/A's enzymatic function. During synthesis of the bead-based immunomatrix it was critically important to use a neutral wash buffer after binding and crosslinking of the antibody to the protein A beads. When an acidic wash buffer (pH 2.8) was used instead, the antibodies were altered such that they became inactive when exposed to nanomolar concentrations of the toxin, probably as a result of toxin-mediated unspecific proteolytic cleavage (Figure S1).

Our assay utilizes a fluorogenic peptide, SNAPtide (U.S. patent 6,504,006, List Biological Laboratories), which is a molecular beacon derivative of SNAP25, the natural substrate of BoNT/A [27], [28]. SNAPtide is cleaved by BoNT/A between a fluorophore and a quencher (a FRET pair), thereby releasing the unquenched fluorophore (Figure 1B and Figure S2C). The SNAPtide used here contains a conjugated fluorescein thiocarbamoyl (FITC) quenched by a 4-(dimethylaminoazo) benzene-4-carboxyl (DABCYL)-moiety. The immobilized polyclonal rabbit antibody used does not inhibit the specific proteolytic activity of BoNT/A and exhibits binding affinity to the light and heavy chain of BoNT/A, as confirmed by Western blot (Figure S2A,B).

### Assay Optimization

**Figure 2:**
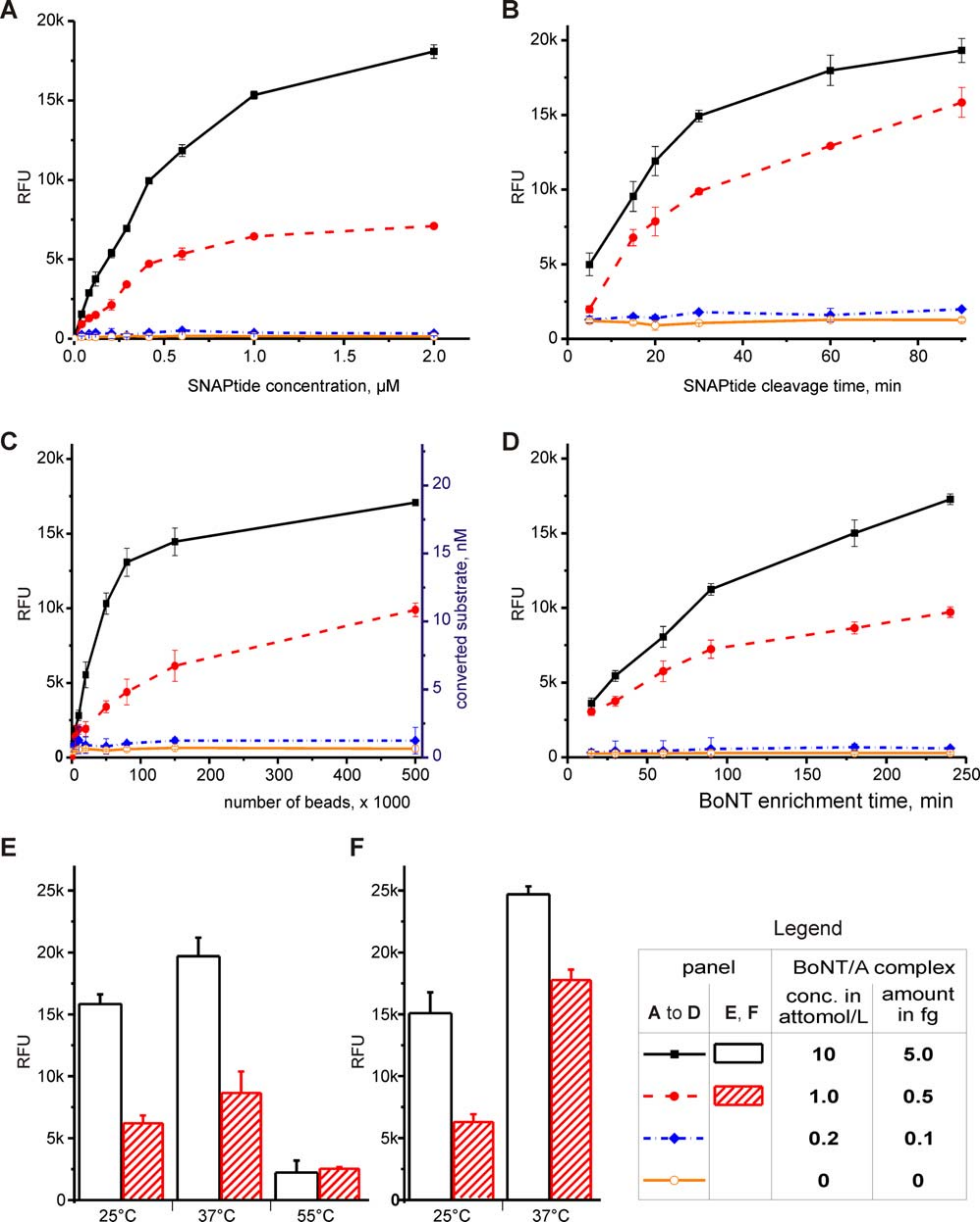
Optimization of the BoNT/A ALISSA in 10% fetal bovine serum. Effect of (A) SNAPtide concentration of SNAPtide after a 1 hr reaction time, (B) SNAPtide cleavage time, (C) number of beads, (D) BoNT enrichment time, (E) temperature during BoNT/A enrichment and (F) temperature on cleavage of the SNAPtide. The following parameters were kept constant unless when varied as indicated: BoNT/A binding and cleavage reaction (25°C); SNAPtide concentration (1 µM); BoNT/A enrichment time and SNAPtide cleavage time (1 hr each); number of beads (120,000).

We optimized the conditions of a prototype bead-based assay to maximize its sensitivity and specificity for one- and ten-attomolar concentrations of toxin in 1-mL sample volumes (Figure 2). Therefore, we repeatedly determined the assay's sensitivity with serial dilutions of BoNT/A in 10% fetal bovine serum (FBS), while evaluating the effect of the following parameters: antibody-to-protein A crosslinking conditions, wash buffers used post-crosslinking of antibodies to beads, number of beads, toxin-antibody binding times and temperatures, wash buffers for removal of molecules that bind non-specifically to the beads, SNAPtide concentration, SNAPtide conversion time and the effect of temperature during the reaction. For each BoNT/A dilution, the fluorescence intensity was plotted against the varying parameter (Figure 2). Due to the asymptotic nature of the resulting curves, there are no optima for several parameters. However, by analyzing the change in signal gain as a function of a given parameter, we identified efficient values for each parameter for which the steepest increase in signal gain has readily been achieved. In consequence, the assay's performance has become robust and predictable.

The assay was efficient and highly sensitive when 100 µL/well SNAPtide in 1 µM-concentration was used (Figure 2A). A reaction time of at least one hour for the conversion of the fluorogenic SNAPtide at room temperature (25°C) was appropriate (Figure 2B). Within limits, the signal-gain of the assay could be enhanced by increasing the number of beads mixed with the sample (Figure 2C) and by extending the enzyme enrichment time (Figure 2D). The most efficient bead concentrations were between 100,000 and 120,000 beads/mL, which correspond to a bead-bed volume of approximately 8.7–10.4 µl when left to settle. Further increase of the bead concentration to 500,000/mL raised the signal intensity by only another 28% (Figure 2C). Sufficient binding of BoNT to the bead-bound antibodies requires 3 hrs at 25°C and just 1 hr at 37°C. The measured toxin activity was diminished at 55°C (Figure 2E), most likely due to toxin deactivation^44^ as opposed to decreased antibody binding. An increase in temperature from 25°C to 37°C during the SNAPtide-conversion reaction also improved the signal somewhat (Figure 2F), and reliable readings were obtained for 1 hour reaction times.

### Assay performance

**Figure 3:**
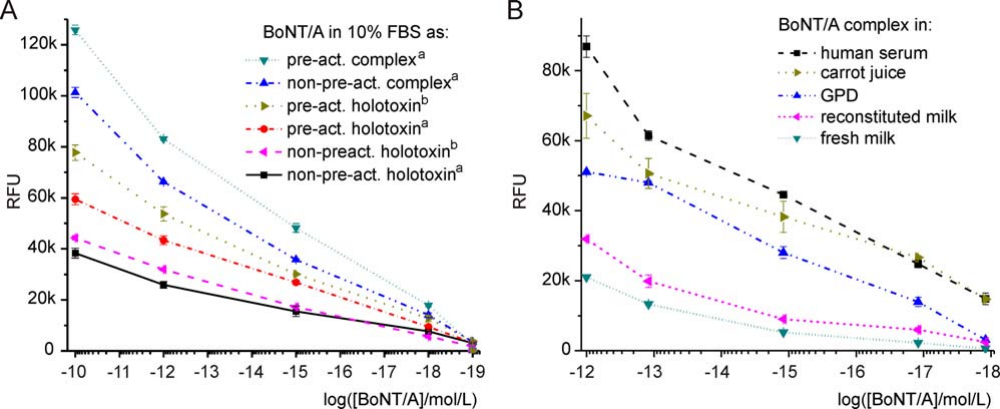
Sensitivities of BoNT/A ALISSA. BoNT/A detection in (A) 10% fetal bovine serum (FBS) to various forms and sources of the toxin; ^a ^Metabiologics (Madison, WI) and ^b ^List Biological Laboratories (Campbell, CA); (B) in undiluted human serum, 50% carrot juice (with binding buffer), gelatin phosphate diluent (GPD), reconstituted powdered milk (fat free) and fresh whole milk (2% fat). Pre-act. = toxin preactivated with 5 mM DTT; all reactions with SNAPtide were carried out with 1.25 mM DTT.

The pre-activated toxin, obtained through short incubations with 5 mM DTT, produced slightly higher signals when compared to the non-pre-activated toxin (Figure 3A). However, the subsequent reaction with the fluorogenic reporter had to be performed in 1.25 mM DTT, in order to avoid denaturation of the immunoaffinity matrix and because prolonged exposure to the more concentrated reductive agent considerably inactivated the toxin considerably (tested on bead-free toxin; data not shown).

#### Sensitivity

The 150-kDa BoNT/A holotoxin (from two different commercial sources) and the 500-kDa type A toxin complex were serially diluted and tested by BoNT/A ALISSA in 10% FBS (Figure 3A). Robust signals of several thousand relative fluorescence units (RFU) above background were still observed for concentrations of one attomol/L toxin in 1-mL sample volumes. Signals for the toxin complex were always stronger than for identical molar concentrations of holotoxin. The practical detection limit in 10% FBS is 1 attomol/L, which corresponds to 0.5 fg of the 500-kDa toxin complex in a 1-mL sample (Figure 2 and 3). For a comparison with other assays see Table 1. For practical reasons, we had used diluted FBS to optimize our assay. To test if the ALISSA can be utilized in other more relevant complex samples, we determined the assay's sensitivity for the toxin complex in spiked undiluted human serum, carrot juice, reconstituted non-fat powdered milk, fresh milk and in GP-diluent (Figure 3B). GP-diluent is used in the mouse bioassay for BoNT detection. Although the discernible fluorescence was less intense than in toxin-spiked samples with 10% FBS, BoNT/A was still detected for 1 attomol/L toxin complex, with signal intensities of ∼14,800, ∼14,750, ∼3,100, ∼2500 and ∼650 RFU above background in undiluted human serum, 50% carrot juice, GP-diluent, non-fat milk and fresh milk, respectively. Overall, the ALISSA signals correlated proportionally with the toxin concentration over several orders of magnitude (Figure 3).

#### Specificity and kinetics

**Figure 4:**
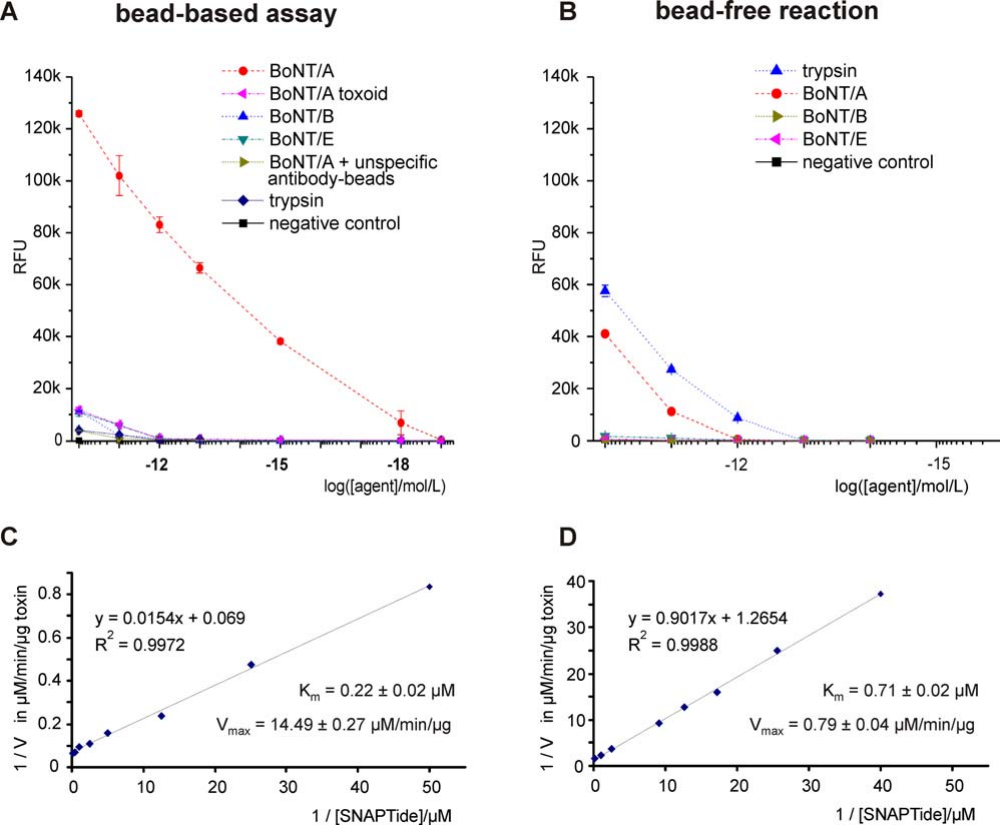
BoNT/A ALISSA versus bead-free SNAPtide cleavage. Comparison between (A, C) the bead-based assay and (B, D) the bead-free reaction in 10% FBS. (C, D) Lineweaver-Burk (double reciprocal) plots of the BoNT/A reaction rate as a function of the substrate (SNAPtide) concentration. Note different y-axis scales. The solid lines represent linear regression fits.

We evaluated specificity and also compared the sensitivity and kinetics of the bead-based ALISSA to those of the bead-free conversion of the reporter peptide. To test the effect of non-specific agents, we used FBS samples mixed with: 1) beads conjugated to purified unspecific rabbit IgG; 2) trypsin, because it can also cleave SNAPtide, but it cannot be enriched on the beads; 3) BoNT complex type B; 4.) BoNT complex type E; 5) type A toxoid, which is a non-toxic, antibody-binding formaldehyde inactivated derivative of BoNT/A; and 6) a toxin-free control (Figure 4A,B). The bead-based assay produced low intensity signals with the non-specific agents (trypsin, BoNT/A toxoid, BoNT/B and E), and only at the highest tested concentrations (10–100 pmol/L). The bead-free reaction mixture produced signals only with trypsin and with BoNT/A for concentrations of 1 pmol/L or greater, and these signals were weak. Surprisingly, in the bead-free reaction, equimolar trypsin concentrations produced even stronger signals than the BoNT/A complex. This was not the case in the bead-based assay. Toxin complexes of type B and E, for which the SNAPtide substrate does not contain specific cleavage sites, produced signals that were nearly undetectable at the 10 and 100 picomolar concentrations. The intensities of these type B and E signals were even lower than the intensities of the signals obtained with the bead-based assay (Figure 4A,B). It is noteworthy that the bead-based detection of BoNT/A produced much higher signals, at any given toxin concentration, than did the bead-free reaction mixture. Discernible signals for the bead-free reaction mixture were obtained only for BoNT/A concentrations greater than or equal to 1 pmol/L. In contrast, strong signals were obtained with BoNT/A at concentrations as low as 1 attomol/L the bead-based assay used, suggesting that the bead-based assay is at least a million-fold more sensitive than the bead-free assay. This remarkable enhancement of the substrate cleavage reaction, when BoNT/A was immobilized on the beads, prompted us to determine the kinetic parameters of the SNAPtide conversion reaction. For comparative purposes, a fixed concentration of BoNT/A complex (100 pmol/L) was used in 1-mL sample volumes for both the reactions with the bead-free toxin and with the bead-immobilized toxin. At this concentration, BoNT/A was easily detected with either method. The hydrolysis of SNAPtide by BoNT/A obeyed Michaelis-Menten kinetics and was characterized by a linear relationship between the reciprocal substrate concentration and the activity of the enzyme (Figure 4C,D). The K_m_ of the immobilized enzyme was 3.2-fold lower than for the free enzyme, indicating a somewhat higher enzyme/substrate affinity. The main effect of binding of the toxin to the beads was found in the rate of catalysis: The immobilized BoNT/A hydrolyzed the SNAPtide substrate at a maximal conversion rate that was 18-fold greater than that of the free toxin. For the free (non-immobilized) and the immobilized enzymes the corresponding values for V_max_ at 25°C were 0.79±0.04 µM/min/µg and 14.49±0.27 µM/min/µg, respectively.

#### Comparison between BoNT/A ALISSA and the mouse bioassay

We performed a direct comparison of the BoNT/ALISSA with the live-mouse bioassay. Both assays used a freshly prepared dilution series of a common stock solution of BoNT/A toxin complex in GP-diluent and were started within a few minutes of the same time point. The ALISSA results became available after ∼2.5 hours, while the mice were observed for the full four days of the standardized bioassay [44], [45] (Table 2). The mouse assay was positive for the highest test concentrations of 300 and 60 pg/mL (0.5 mL injected per mouse). Mild symptoms of botulism developed within 96 hours in three of five mice that received one 1/100 of the mouse LD_50_ (0.3 pg). The mice that received 10^−4^ or 10^−5^ LD_50_ remained completely asymptomatic. BoNT/A ALISSA produced robust, clear signals throughout the entire series of toxin dilutions. At the lowest test concentration (0.6 fg/mL [10^−5^ LD_50_]) the ALISSA fluorescence signal was still ∼3,100 units above background (Table 2).

**Table 2:** Comparison of the mouse bioassay with BoNT/A ALISSA.

[toxin A complex] (fg/mL)	LD_50_s*^a^*	Mouse bioassay result	ALISSA result (RFU)
300,000.0	5	5/5 dead in <4 hrs	51,105±95
60,000.0	1	5/5 dead in <21 hrs	48,009±464
600.0	10^−2^	3/5 mild symptoms*^b^*	28,049±1713
6.0	10^−4^	5/5 disease free*^b^*	13,954±1324
0.6	10^−5^	5/5 disease free*^b^*	3,116±15
0.0	0	n.d.	0±8

*^a^* calculated per injected 0.5 mL sample; one LD_50_ = 30 pg BoNT/A complex; *^b^* all mice alive after 96 hrs; n.d., not determined; sample medium = gelatin phosphate diluent.

## Discussion

The BoNT/A ALISSA avoids interference with other sample components by using a highly BoNT/A-specific affinity matrix and by using a BoNT/A-specific substrate to exploit the natural proteolytic activity of the toxin. Both steps also amplify the signal by 1) localized enrichment of the toxin and 2) through enzymatic conversion of billions of substrate molecules per toxin molecule. The capture matrix is designed to stably enrich the toxin, while retaining its enzymatic activity and by purifying the toxin from other unspecific, sample-contained, proteases. Like the Endopep-MS method [36]–[39], we used a beaded protein A matrix to bind anti-BoNT/A antibodies via their FC-portions, orienting the antibody binding domains away from the bead surface and into the surrounding fluid, which we expected to provide high accessibility for toxin molecules.

The plateaus seen in the assay's response curves, used to optimize the substrate concentration (Figure 2A) and the size and volume proportions of the immunosorbent matrix (Figure 2C), represent saturation effects that indicate when the substrate concentration is no longer rate-limiting or how much antibody is needed to capture most of the toxin molecules, respectively. We have determined that the antibody binding capacity of the beads was 50 µg antibody per one million beads, and therefore, we estimated the antibody's dissociation constant k_D_ at half maximum saturation to be approximately 15 nM (Figure 2C). It should be possible to further improve the assay's sensitivity by using antibodies with higher binding affinities. High affinity anti-BoNT antibodies have also been used as antitoxins to neutralize systemic botulinum toxin in botulism patients [46], [47]. It is important to point out that this mode of “neutralization” should not be confused with inactivation of the toxin's enzymatic activity by steric hindrance of the catalytic site as a result of antibody binding. The antibody-mediated “neutralization” of the toxin in the human body relies on the formation of an antibody-antigen complex and its subsequent hepatic accumulation and clearance [48], [49].

The use of a standard curve to measure the concentration-dependent intensity of the fluorescence signal of an un-quenched calibration peptide (Figure S3) enabled us to determine the molar conversion rate for the substrate molecules. For the 10 attomolar toxin concentration, we calculated a substrate conversion rate of approximately two billion substrate molecules per one immobilized toxin molecule per hour. The reaction is limited by the rate at which the toxin becomes inactivated. Several factors may contribute to the inactivation of the toxin: 1) chelation of the zinc atom [50] by DTT, 2) denaturation of the toxin by the reducing buffer [25], or 3) proteolytic degradation of the toxin either through autoproteolysis [51], [52] or by a contaminant protease.

The optimal temperature for the assay is 37°C (Figure 2E,F), which coincides with the temperature at which the natural action of the toxin occurs [53] and at which binding to the IgG antibody appears to be optimal. Higher temperatures inactivate the toxin [53], [54]. The pH of the sample should be approximately neutral (between 6 and 8). Further improvement of the assay's sensitivity may be feasible by reducing the background fluorescence of the uncleaved SNAPtide. The FITC/DABCYL donor/acceptor pair used in this study already exhibits some fluorescence due to a non-optimal overlap of the FITC emission spectrum with the absorption spectrum of DABCYL (Figure S4). A peptide-conjugated FRET pair with a 2,4-dinitrophenyl acceptor and a 4-methyl-7-dimethylamino-coumarin donor has been described as a substrate for BoNT/A [55]. This and other FRET pairs with a better spectral overlap, potentially lower background fluorescence, and good kinetic properties may be tested as fluorogenic substrates in the future.

Our kinetic studies revealed an approximately 18-fold increase in the maximum conversion rate v_max_ and a three-fold higher affinity to the substrate (three-fold lower k_M_) for the immobilized toxin than for the free toxin in solution (Figure 4C,D). Similar improvements in enzymatic activity have been reported for other immobilized enzymes, such as immobilized trypsin, and the effect is attributed to the increased reaction surface area and the control of diffusion through more frequent substrate-enzyme interactions[56]. We have also detected a weak interaction between the uncleaved substrate and the bead surface (Figure S5). This weak interaction may contribute to a higher local concentration of SNAPtide on the bead surface and a shorter time of diffusion along the bead surface for a SNAPtide molecule to encounter the active site of a toxin molecule.

The average bead surface area in the ALISSA assay is approximately 7.85 cm^2^ per sample (based on a 50 µm average bead diameter) whereas the antigen-binding surface area in a conventional ELISA with a 96-well flat-bottom polystyrene microplate measures only about 0.256 cm^2^ per well (BD Falcon plate, cat. #353279). The available reaction surface area in the ALISSA is therefore about 30-fold larger than in an ELISA. In addition, it is possible that immobilized BoNT/A is better protected from proteolysis and aggregation. Once immobilized, molecules of the unstable BoNT/A light chain should be sufficiently separated from each other to diminish their autocatalytic degradation. The use of a bead-based assay allows for stringent wash steps that diminish interference with other proteases, as demonstrated for BoNT/A in comparison with equimolar concentrations of trypsin, BoNT/B and BoNT/E.

The ALISSA performed with comparable sensitivities in undiluted human serum, 50% carrot juice (adjusted to pH 7.5 with binding buffer), reconstituted powdered milk, fresh milk and GP-diluent. In direct comparison with the mouse assay the ALISSA was considerably faster and four to five orders of magnitude more sensitively (Table 2.). A more accurate comparison would require a significantly larger number of mice to obtain a precise mouse-LD_50_ (MLD_50_). Here we used the MLD_50_ provided by the toxin manufacturer, and found that the value given was largely consistent with our experimental results. Ultimately, the BoNT ALISSA might help to reduce animal use for the associated lethal test procedure. A rough estimate on the economy of the assay reveals an average reagent cost of ∼$15 per sample (Table S1), which is expected to be much lower than the costs associated with utilization of an animal facility to ensure constant availability of live animals for diagnostic purposes.

### Conclusion

The BoNT/A ALISSA is an inexpensive, simple and robust procedure that ensures high analytical specificity and attomolar sensitivity for the detection of BoNT/A in complex biological samples. Future modification of our assay to encompass other BoNT serotypes, and even other classes of toxins, should become feasible as soon as the necessary substrate reagents become available.

## Materials and Methods

### Reagents

The 150-kDa BoNT A holotoxin was purchased from List Biological Laboratories (Campbell, CA) and from Metabiologics Inc. (Madison, WI). The 500-kDa BoNT/A complex and the BoNT/A toxoid were obtained from Metabiologics. All toxins were produced by a Hall A strain of *Clostridium botulinum*. The BoNT/A subtype used here is therefore A1. Toxin activities for the holotoxin and the complex were 2.1×10^8^ MLD_50_/mg and 3.6×10^7^ MLD_50_/mg, respectively, according to Metabiologics. The fluorogenic peptide SNAPtide (FITC/DABCYL, product #521) and the unquenched calibration peptide, containing an N-terminally FITC-labeled fragment of SNAPtide (product #528, synthetic, but sequence identical to the BoNT/A cleaved product), were from List Biological Laboratories. Affinity purified rabbit polyclonal antibodies against *Clostridium botulinum* A toxoid were from Abcam (Cambridge, MA, cat #ab20641). Purified rabbit IgG was from ICN Pharmaceuticals, Inc. (Aurora, OH), the Seize X Protein A immunoprecipitation kit was from Pierce (Rockford, Il), trypsin was from Promega, and fetal bovine serum was from Invitrogen (Carlsbad, CA). Human serum was from Sigma (cat. #H4522) and carrot juice was from Bolthouse Farms (Organics, 100% carrot juice, 1 liter bottle). Other reagents were from Sigma unless indicated.

### Gel electrophoresis and Western Blots

Gels were analyzed by Western Blot using the same polyclonal rabbit antibody that was used in the ALISSA. Gels bands were visualized with the SimpyBlue SafeStain kit from Invitrogen (Carlsbad, CA).

### Preparation of the immunomatrix

Anti-BoNT/A antibodies were bound and then covalently cross-linked to the bead-immobilized protein A molecules to prevent them bleeding off when mixed with the sample. The release of antibodies into the sample would otherwise lower the sensitivity of the assay drastically. To produce such a stable immunomatrix we used a Seize X Immunoprecipitation Kit (Pierce, product #45215) essentially as described by the manufacturer, but with some modifications. Briefly, 125 µg of anti-BoNT/A antibodies in 0.4 mL of the kit's binding/wash buffer (14 mM NaCl, 8 mM sodium phosphate, 2 mM potassium phosphate and 10 mM KCl, pH 7.4) were incubated with the protein A bead support for one hour to ensure complete binding. Crosslinking was performed by adding 25 µL of freshly dissolved disuccinimidyl suberate (DSS) (25 µg/µL) in DMSO to the beads in a Handee spin cup followed by gentle mixing on a Labquake rotisserie (Barnstead International, Dubuque, IA) for 30-60 minutes at room temperature. The beads were centrifuged and washed three times with 500 µL “Gentle Elution buffer” and two times with 500 µL “Gentle Binding” buffer (Pierce, product #21030). Spin Cup columns with functionalized beads were wrapped with parafilm and stored at 4°C until used. Bead numbers were enumerated with a Reichert Bright-Line Metallized Hemacytometer from Hausser Scientific (Horsham, PA).

### BoNT/A assay with a large immuno-sorbent surface area (ALISSA)

The concentrations of the substrate and the amounts of time for BoNT/A enrichment the cleavage reaction were varied as shown in Figure 2; the values presented here are the optimized values. BoNT/A holotoxin or BoNT complex type A were reconstituted (when received dry) in 20 mM HEPES, pH 8.0, 0.05% Tween-20, 0.3 mM ZnCl_2_, 1.0 mg/mL BSA. The toxin stock concentrations were validated by two independent methods 1) Bicinchoninic acid protein assay kit (Pierce) and 2) UV photometric by measuring of the absorbance at 280 nm with a NanoDrop ND-1000 system (Thermo Fisher). The toxin solutions were either used untreated or pre-activated by incubating with 5 mM dithiothreitol (DTT) for 30 minutes at 37°C. Serial dilutions of BoNT/A, B, E and trypsin in concentrations from nanomolar to subattomolar were prepared separately in 2.0 mL tubes with either 10% fetal bovine serum (FBS), undiluted human serum, 50% carrot juice (in “Gentle Binding” buffer, pH 7.5), 3% reconstituted non-fat milk (Carnation from Nestle) in Dulbecco's phosphate buffered saline solution (PBS) (Irvine Scientific, Santa Ana, CA), fresh milk (2% fat), or gelatin phosphate diluent (2 g/L gelatin (Difco, cat #214340), 4 g/L dibasic sodium phosphate, pH adjusted with 1 M HCl to 6.2). For the comparison of bead-free versus bead-based assay, toxin concentrations and total toxin amounts were identical in each dilution step. Beads with immobilized antibodies were added to the toxin-spiked samples and rotated at 8 rpm for 1 hour at 37°C, centrifuged at 1700 g for 10 min, and washed three times with 500 µL PBS buffer. A fat-solubilizing wash buffer (PBS with 40 mM HEPES) was required for analyses of fresh milk samples. The beads were resuspended in 300 µL reaction buffer (20 mM HEPES, 0.3 mM ZnCI_2_, 1.25 mM DTT, 0.1% Tween-20, pH 8.0) with 1.0 µM SNAPtide allowed to react in the dark for 1 hour at 37°C while rotating at 8 rpm. The tubes were centrifuged and the supernatant was distributed evenly in triplicates (100 µL/well) on a black, polystyrene, 96-well flat-bottom microtiterplate (Fisher Scientific) and mixed with 200 µL EDTA (20 mM). Fluorescent intensities were measured on a Wallac 1420 Multilabel Counter Victor^2^ spectrophotometer (PerkinElmer) at excitation and emission wavelengths of 485 nm and 535 nm, respectively.

### Microscopy

Beads were mounted on glass slides and examined in phase contrast and fluorescence modes with an Olympus BH2-RFCA microscope (Japan).

### Kinetic studies

K_m_ and V_max_ values for the reactions of BoNT/A with bead-free or bead-immobilized SNAPtide were calculated from double reciprocal Lineweaver–Burk plots. Eight distinct concentrations of substrate from 0.025 to 5 µM were used. Initial velocities for the reactions were calculated from linear regression analysis as µM of cleaved SNAPtide/min/mg enzyme. Values are averages of four independent determinations.

### Comparison of BoNT/A ALISSA with the mouse assay

A split aliquot of 100 ng BoNT/A toxin complex was shipped overnight in a refrigerated hazmat container from City of Hope to the Infant Botulism Treatment and Prevention Program of the California Department of Public Health (CDPH) in Richmond for use in the diagnostic live-mouse bioassay. Identical dilution series of the toxin in GP-diluent were prepared concurrently in pre-prepared and weighed vials at both institutions. 18-22 g mice were injected i.p. with 0.5 mL/mouse of sample and watched for signs of botulism or death for the standard 96 hour observation period [44]. The start of the i.p. mouse injections at CDPH were coordinated by telephone to coincide with the start of the ALISSA at City of Hope within a margin of minutes. BoNT/A ALISSA results became available after ∼2.5 hours.

## Supporting Information

Figure S1 The capture antibodies are altered when the beads are washed with an acidic buffer (pH 2.8) following IgG-to-protein A binding and crosslinking. As a result the relative fluorescence intensity of the assay is reduced in presence of BoNT/A at concentrations >50 pg/mL, presumably due to unspecific degradation of the antibodies by the metalloprotease.
(0.03 MB PDF)

Figure S2 (A) Reaction of the fluorogenic SNAPtide with BoNT/A in the absence or presence of an anti-BoNT antibody (in molar excess) in 10% FBS. (B) SDS gel bands and Western blot of the BoNT/A complex. The Western blot was developed with the rabbit anti-BoNT/A antibody used for ALISSA. (C) Micrograph of a bead particle used to immobilize BoNT/A in phase contrast (left) and under fluorescence (right) after reacting with SNAPtide. RFU = relative fluorescence units; HC, LC = BoNT/A heavy and light chain, respectively.
(0.13 MB PDF)

Figure S3 Standard curve of the fluorescence signal of the unquenched calibration peptide, which is structurally identical to the FITC-containing cleavage product resulting from BoNT/A hydrolysis of SNAPtide by BoNT/A; y in RFU; x in nM; R is the correlation coefficient
(0.03 MB PDF)

Figure S4 UV/VIS spectra of the quencher DABCYL and the fluorophore FITC. Absorbance spectrum of DABCYL (green dashed line), emission spectra of FITC as an fluorescein antibody conjugate (blue line) and as a fluorescein-dextran conjugate (red line), both at pH 8.0; adapted from the online Fluorescence Spectra Viewer (http://probes.invitrogen.com/servlets/spectra/) with kind permission from Dr. Iain Johnson, Invitrogen Corporation, Molecular Probes.
(0.18 MB PDF)

Figure S5 Weak interaction of SNAPtide with protein A sepharose beads: One mL of 50 nM SNAPtide was incubated with 0, 70 k, 140 k and 350 k beads in the reaction buffer for 1 hour at 37°C. The supernatant was removed and treated with trypsin, resulting in approximately 10% cleavage of SNAPtide. A ∼30% reduction in the fluorescence signal is observed for 350,000 beads, which corresponds to the absorption/adsorption ratio of ∼30% of the original SNAPtide and reflects a 10-fold concentration of SNAPtide within the bead volume of 30 µL.
(0.03 MB PDF)

Table S1 ALISSA cost analysis
(0.03 MB DOC)
