# Supplementary material for: Attomolar Detection of Botulinum Toxin Type A in Complex Biological Matrices
Source: PLoS One. 2008 Apr 30;3(4):e2041. doi: 10.1371/journal.pone.0002041 (PMC2323579; doi:10.1371/journal.pone.0002041)
Supplement: Figure S1 — The capture antibodies are altered when the beads are washed with an acidic buffer (pH 2.8) following IgG-to-protein A binding and crosslinking. As a result the relative fluorescence intensity of the assay is reduced in presence of BoNT/A at concentrations >50 pg/mL, presumably due to unspecific degradation of the antibodies by the metalloprotease. (0.03 MB PDF) [file pone.0002041.s001.pdf]

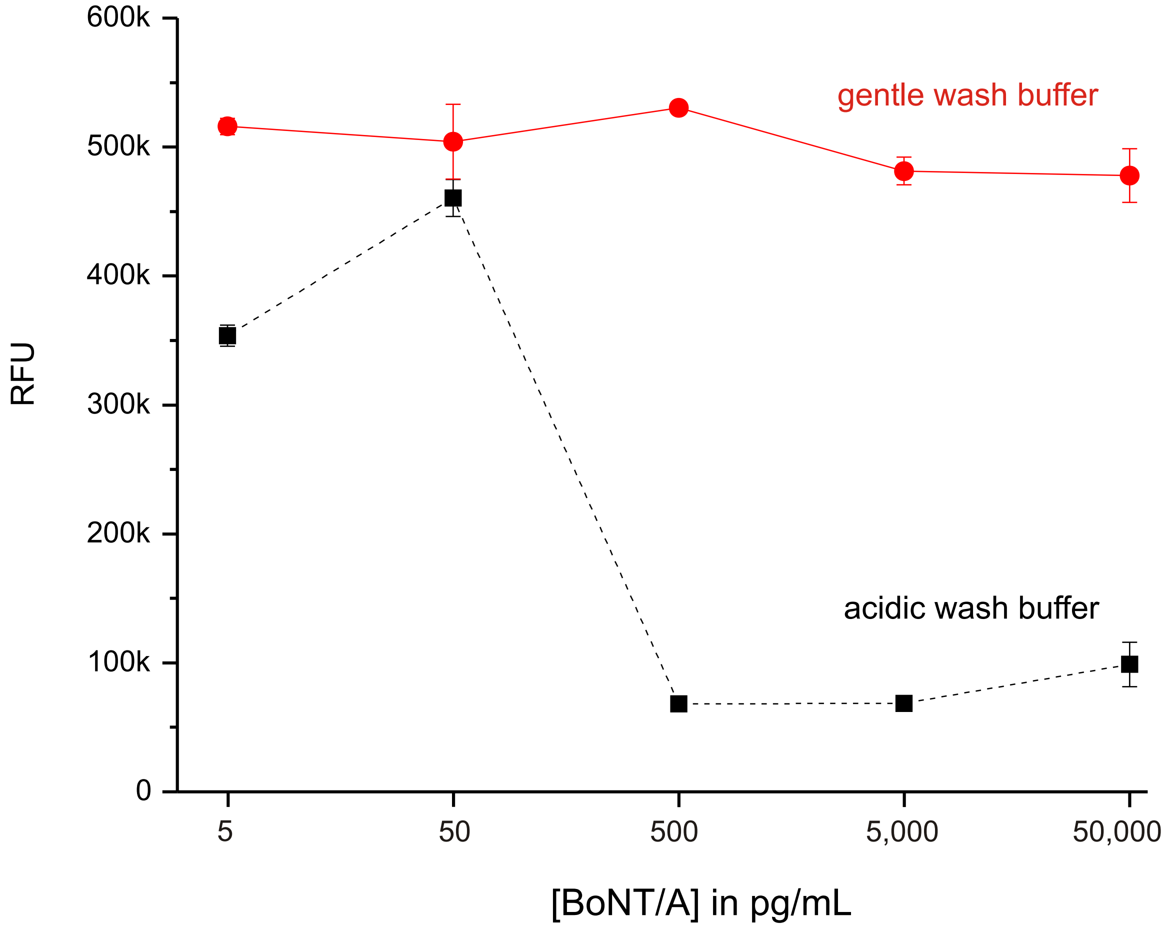

Figure S1: The capture antibodies are altered when the beads are washed with an acidic buffer (pH 2.8) following IgG-to-protein A binding and crosslinking. As a result the relative fluorescence intensity of the assay is reduced in presence of BoNT/A at concentrations > 50 pg/mL, presumably due to unspecific degradation of the antibodies by the metalloprotease.
