# Supplementary material for: Attomolar Detection of Botulinum Toxin Type A in Complex Biological Matrices
Source: PLoS One. 2008 Apr 30;3(4):e2041. doi: 10.1371/journal.pone.0002041 (PMC2323579; doi:10.1371/journal.pone.0002041)
Supplement: Figure S2 — (A) Reaction of the fluorogenic SNAPtide with BoNT/A in the absence or presence of an anti-BoNT antibody (in molar excess) in 10% FBS. (B) SDS gel bands and Western blot of the BoNT/A complex. The Western blot was developed with the rabbit anti-BoNT/A antibody used for ALISSA. (C) Micrograph of a bead particle used to immobilize BoNT/A in phase contrast (left) and under fluorescence (right) after reacting with SNAPtide. RFU = relative fluorescence units; HC, LC = BoNT/A heavy and light chain, respectively. (0.13 MB PDF) [file pone.0002041.s002.pdf]

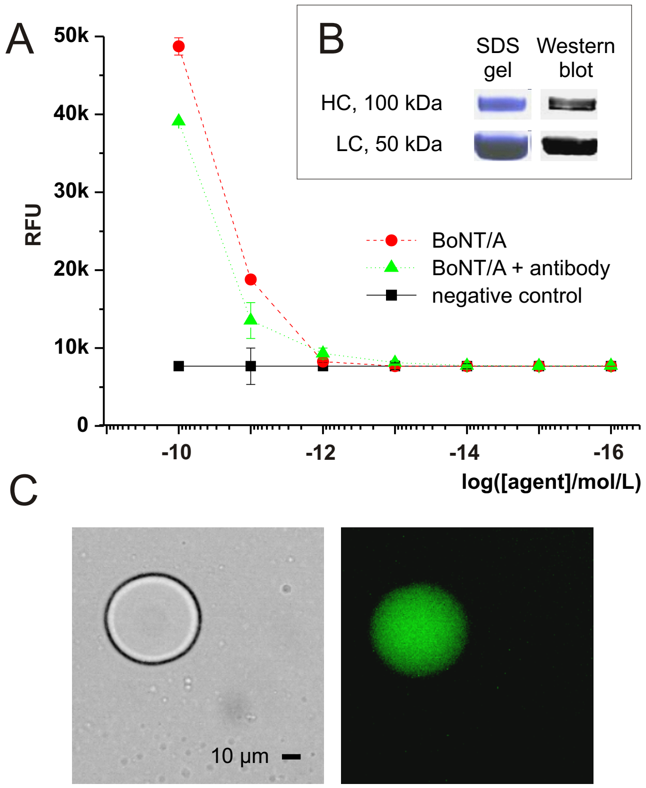

Figure S2: (A) Reaction of the fluorogenic SNAPtide with BoNT/A in the absence or presence of an anti-BoNT antibody (in molar excess) in 10% FBS. (B) SDS gel bands and Western blot of the BoNT/A complex. The Western blot was developed with the rabbit anti-BoNT/A antibody used for ALISSA. (C) Micrograph of a bead particle used to immobilize BoNT/A in phase contrast (left) and under fluorescence (right) after reacting with SNAPtide. RFU = relative fluorescence units; HC, LC = BoNT/A heavy and light chain, respectively.
