# Supplementary material for: Attomolar Detection of Botulinum Toxin Type A in Complex Biological Matrices
Source: PLoS One. 2008 Apr 30;3(4):e2041. doi: 10.1371/journal.pone.0002041 (PMC2323579; doi:10.1371/journal.pone.0002041)
Supplement: Figure S3 — Standard curve of the fluorescence signal of the unquenched calibration peptide, which is structurally identical to the FITC-containing cleavage product resulting from BoNT/A hydrolysis of SNAPtide by BoNT/A; y in RFU; x in nM; R is the correlation coefficient (0.03 MB PDF) [file pone.0002041.s003.pdf]

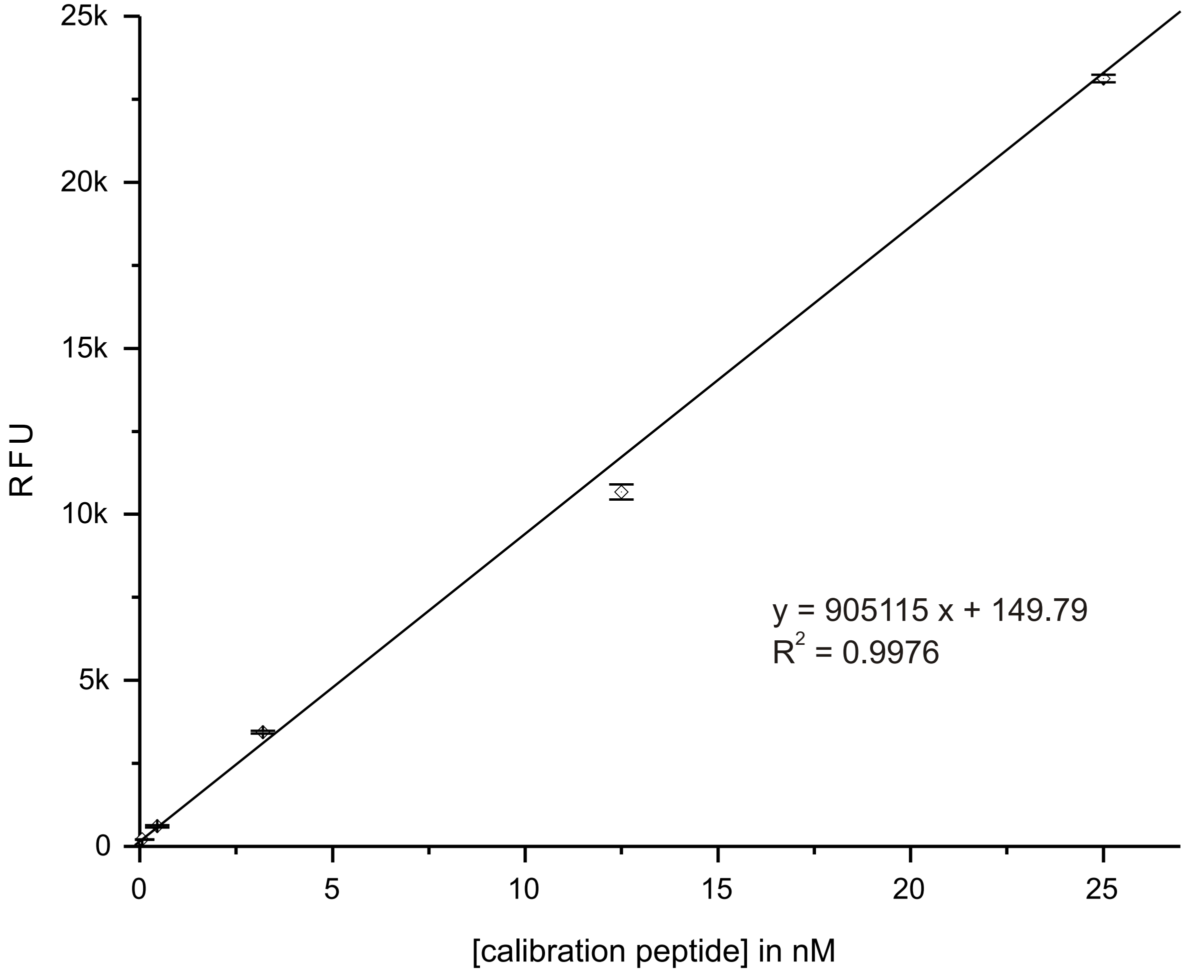

Figure S3: Standard curve of the fluorescence signal of the unquenched calibration peptide, which is structurally identical to the FITC-containing cleavage product resulting from BoNT/A hydrolysis of SNAPtide by BoNT/A; *y* in RFU; *x* in nM; *R* is the correlation coefficient
