# Supplementary material for: Attomolar Detection of Botulinum Toxin Type A in Complex Biological Matrices
Source: PLoS One. 2008 Apr 30;3(4):e2041. doi: 10.1371/journal.pone.0002041 (PMC2323579; doi:10.1371/journal.pone.0002041)
Supplement: Figure S4 — UV/VIS spectra of the quencher DABCYL and the fluorophore FITC. Absorbance spectrum of DABCYL (green dashed line), emission spectra of FITC as an fluorescein antibody conjugate (blue line) and as a fluorescein-dextran conjugate (red line), both at pH 8.0; adapted from the online Fluorescence Spectra Viewer (http://probes.invitrogen.com/servlets/spectra/) with kind permission from Dr. Iain Johnson, Invitrogen Corporation, Molecular Probes. (0.18 MB PDF) [file pone.0002041.s004.pdf]

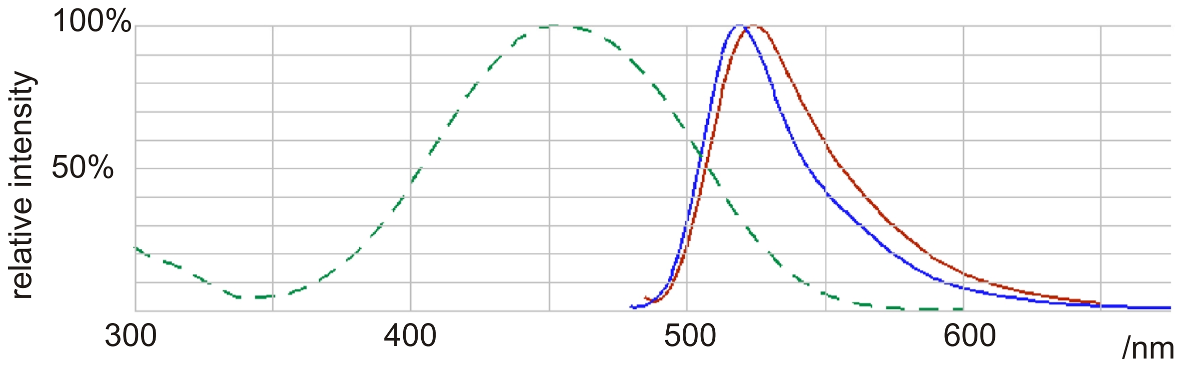

Figure S4: UV/VIS spectra of the quencher DABCYL and the fluorophore FITC. Absorbance spectrum of DABCYL (green dashed line), emission spectra of FITC as an fluorescein antibody conjugate (blue line) and as a fluorescein-dextran conjugate (red line), both at pH 8.0; adapted from the online Fluorescence Spectra Viewer (http://probes.invitrogen.com/servlets/spectra/) with kind permission from Dr. Iain Johnson, Invitrogen Corporation, Molecular Probes.
