# Supplementary material for: Attomolar Detection of Botulinum Toxin Type A in Complex Biological Matrices
Source: PLoS One. 2008 Apr 30;3(4):e2041. doi: 10.1371/journal.pone.0002041 (PMC2323579; doi:10.1371/journal.pone.0002041)
Supplement: Figure S5 — Weak interaction of SNAPtide with protein A sepharose beads: One mL of 50 nM SNAPtide was incubated with 0, 70 k, 140 k and 350 k beads in the reaction buffer for 1 hour at 37°C. The supernatant was removed and treated with trypsin, resulting in approximately 10% cleavage of SNAPtide. A ∼30% reduction in the fluorescence signal is observed for 350,000 beads, which corresponds to the absorption/adsorption ratio of ∼30% of the original SNAPtide and reflects a 10-fold concentration of SNAPtide within the bead volume of 30 µL. (0.03 MB PDF) [file pone.0002041.s005.pdf]

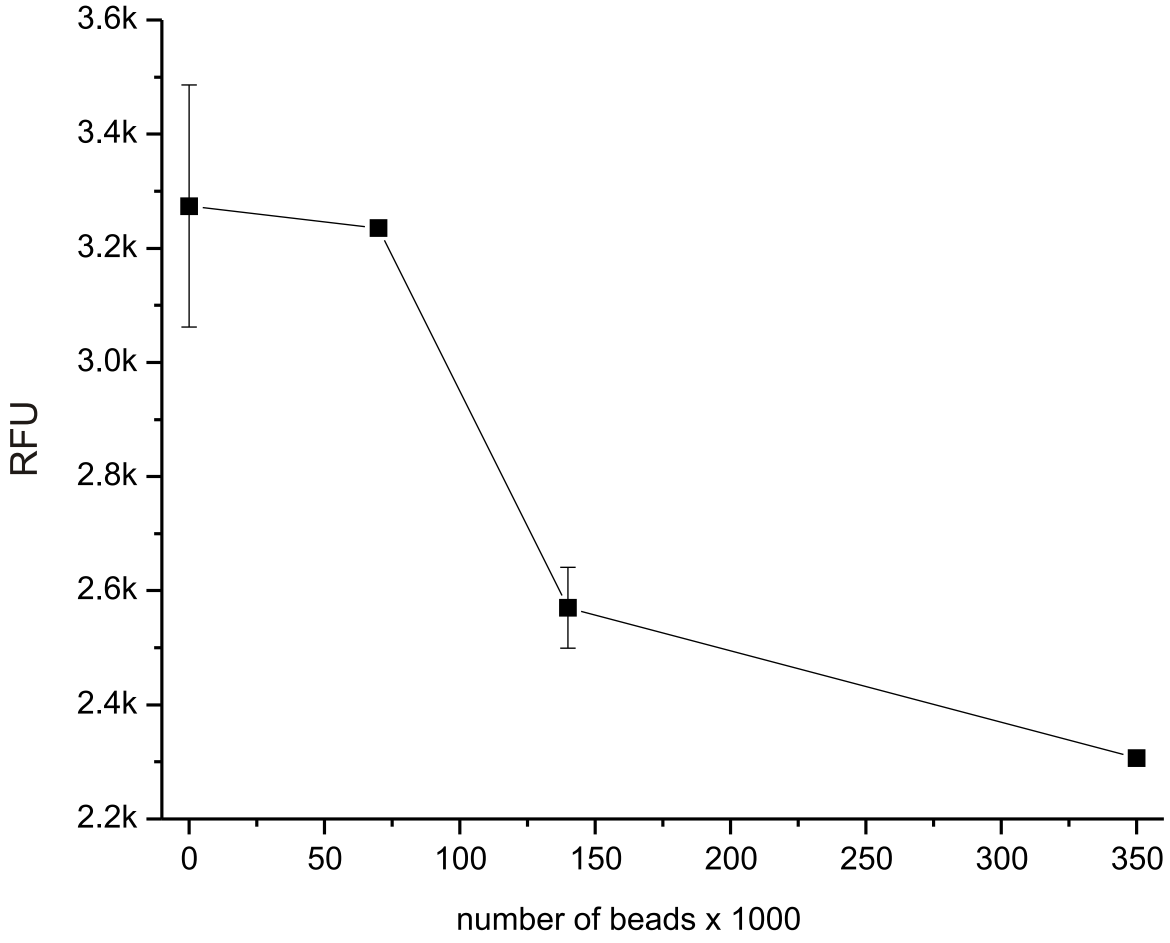

Figure S5: Weak interaction of SNAPtide with protein A sepharose beads: One mL of 50 nM SNAPtide was incubated with 0, 70k, 140k and 350k beads in the reaction buffer for 1 hour at 37°C. The supernatant was removed and treated with trypsin, resulting in approximately 10% cleavage of SNAPtide. A ~30% reduction in the fluorescence signal is observed for 350,000 beads, which corresponds to the absorption/adsorption ratio of ~30% of the original SNAPtide and reflects a 10-fold concentration of SNAPtide within the bead volume of 30 µL.
