# Supplementary material for: Attomolar Detection of Botulinum Toxin Type A in Complex Biological Matrices
Source: PLoS One. 2008 Apr 30;3(4):e2041. doi: 10.1371/journal.pone.0002041 (PMC2323579; doi:10.1371/journal.pone.0002041)
Supplement: Table S1 — ALISSA cost analysis (0.03 MB DOC) [file pone.0002041.s006.doc]

**Table S1 ALISSA cost analysis**

| **Reagent** | **price per unit** | **number of samples per unit** | **price per sample** |
| --- | --- | --- | --- |
| polyclonal rabbit anti-BoNT/A antibody | $327 | 50 | $6.54 |
| Seize X protein A immunoprecipitation kit | $230 | 41 | $5.61 |
| SNAPtide | $377 | 156 | $2.42 |
| black flat bottom microtiter plate | $2 | 96 | $0.02 |
| other reagents (DTT, ZnCl_2_, HEPES, DMSO, Tween-20, PBS, EDTA, etc.) | $250 | 1000 | $0.25 |
| amber polypropylene 2.0mL tubes | $18 | 250 | $0.07 |
| amber polypropylene 0.2mL tubes | $37 | 1000 | $0.04 |
|  |  | **Total:** | **$14.94** |

The cost analysis does not include instrumentation, laboratory equipment, salaries, and taxes
